# Infectivity and Potential Zoonotic Characteristics of Porcine Pseudorabies Virus in Human Cells

**DOI:** 10.1155/2024/5929976

**Published:** 2024-07-16

**Authors:** Xue Li, Nan Li, Jiawei Zheng, Xinru Lv, Yaqi Han, Huimin Zhang, Ying Ren, Gefen Yin, Linzhu Ren

**Affiliations:** ^1^ College of Animal Sciences State Key Laboratory for Diagnosis and Treatment of Severe Zoonotic Infectious Diseases Jilin University, 5333 Xi'an Road, Changchun 130062, China; ^2^ Changchun Veterinary Research Institute Chinese Academy of Agricultural Sciences, 666 Liuying West Road, Changchun 130122, China; ^3^ Public Computer Education and Research Center Jilin University, 5333 Xi'an Road, Changchun 130062, China; ^4^ College of Veterinary Medicine Yunnan Agricultural University, Kunming 650201, Yunnan, China

## Abstract

Pseudorabies virus (PRV) is widely spread, characterized by high contagiousness, high viral load, and strong infectivity, and poses severe threats to the global pig farming industry. Apart from pigs, PRV can also infect several other mammals, including mice, cattle, cats, dogs, and wolves, with diverse clinical symptoms. Notably, approximately more than 20 cases of human PRV infection have been reported in recent years, with fever, seizures, human encephalitis, intraocular inflammation, and severe central nervous system symptoms. However, whether PRV can infect humans or belongs to a zoonotic virus is still controversial. In this study, human neuronal cells were infected with PRV and blindly passaged to obtain human cell-adapted PRV, followed by comparing the characteristics of human cell-adapted PRV and pig-derived PRV *in vitro* and *in vivo*, to determine whether PRV has the potential to infect humans. The results showed that PRV could be stably passaged in human cells and produced progeny viruses similar to the parental virus, including morphology, infectivity, and pathogenicity. The human cell-adapted PRV can also cross-transmit to cells from other origins, including humans, mice, pigs, and monkeys, causing different cytopathic effects. Moreover, multiple tissue damage can be detected in mice infected with human cell-adapted PRV. These results demonstrate that PRV is a potential zoonotic virus, and it is necessary to pay close attention to the spread and variation of the virus in animals and humans.

## 1. Introduction

Pseudorabies virus (*Suid herpesvirus 1*, or *Aujeszky's disease virus*, PRV) belongs to the genus *Varicellovirus*, subfamily *Alphaherpesvirinae* of the *Herpesviridae* family [1]. PRV infection is characterized by high contagiousness, high viral load, and strong infectivity. The main routes of PRV transmission include direct contact, droplet spread, air aerosol spread, contaminated items, and so on [2, 3, 4]. The virus spreads rapidly within pig farms, causing widespread infections and triggering acute infectious diseases in pigs. PRV infection can occur in pigs of different age groups, but weaned and fattening pigs are most susceptible [5, 6,7]. After PRV infection, the virus rapidly replicates *in vivo*, causing viral dissemination in the bloodstream and further triggering symptoms in multiple systems, such as mental abnormalities, tremors, muscle stiffness, paralysis, respiratory distress, diarrhea, and vomiting. The course of the disease usually lasts 1–3 weeks, with a relatively high mortality rate [8]. In pregnant sows, PRV infection may lead to reproductive disorders, such as abortions, stillbirths, and deformed fetuses [9, 10]. PRV is widely spread globally and has been reported in multiple regions, including the United States, Europe, and Asia, which poses a severe threat to the global pig farming industry, causing significant economic losses [4, 6, 11, 12].

Research on PRV transmission mainly focuses on its transmission capability, routes, and genetic evolution. Research on this virus's transmission pathways, disease course, and severity is also limited. Therefore, it is urgent to closely monitor its transmission situation and strengthen prevention and control measures. Apart from pigs, PRV can infect several other mammals, such as mice, cattle, cats, dogs, and wolves, with clinical symptoms including fever, difficulty breathing, diarrhea, and neurological symptoms [13, 14, 15, 16, 17]. Therefore, the mechanism of PRV cross-species transmission and host range still needs further study. Notably, approximately more than 20 cases of human PRV infection have been reported in recent years, with fever, seizures, human encephalitis, intraocular inflammation, and severe central nervous system symptoms [13, 18, 19, 20, 21, 22, 23, 24]. However, the transmission and infection of PRV in humans are poorly understood, and there are few case reports of PRV cross-species transmission in humans.

In this study, human neuronal cells were infected with PRV and blindly passaged several times to obtain human cell-adapted PRV, followed by comparing the characteristics of human cell-adapted PRV and pig-derived PRV *in vitro* and *in vivo*, to determine whether PRV has the potential to infect humans. The result will help us further understand the biological characteristics and infectivity of PRV, as well as its potential for cross-species transmission, which may provide references for formulating more effective prevention and control measures to prevent the spread and expansion of related epidemics.

## 2. Materials and Methods

### 2.1. Cells and Virus

Human cells (A549, WI38, HepG2, HEH2, HUVEC, HSAS4, SH-SY5Y, and HEK293), porcine cells (PK-15 and ST), murine microglia (BV2) and mouse neuroblastoma-2a (N2a) cells, and monkey cells (Vero) were stored in our lab and used in this study.

HEK293, HepG2, WI38, HUVEC, HSAS4, A549, N2a, and ST cells were cultured in DMEM (Gibco, USA) with 10% fetal bovine serum (FBS). SH-SY5Y cells were cultured in DMEM/F-12 (Gibco, USA) containing 10% FBS. BV2 was cultured in RPMI 1640 (Gibco, USA) with 10% FBS, and Vero and PK15 cells were cultured in DMEM (Gibco, USA) with 5% FBS. All cells were cultured at 37°C in a conventional humidified incubator containing 5% CO_2_.

The PRV JL-21 strain was previously isolated from the renal tissue sample of diseased pigs in Jilin province, China [25]. And the strain was stored in our lab.

### 2.2. Three-Dimensional> (3D) Cultures of SH-SY5Y and HEK293 Cells

3D spheroids were generated under the same culture conditions as the 2D cell culture except using ultralow attachment surface polystyrene 24-well plates (Corning Inc., Corning, NY, USA) as a culture system. In brief, SH-SY5Y and HEK293 cells were cultured in 100 mm dishes following the same procedure as the 2D cell culture, respectively. Upon reaching approximately 90% confluence, the cells were detached using 0.25% trypsin/EDTA after washing with phosphate-buffered saline (PBS) and resuspended in the 1 mL growth medium. The cells were transferred into ultralow attachment surface polystyrene 24-well plates for 24 hr to form 3D spheroid cultures.

### 2.3. Virus Infection In Vitro

PK15 cells were infected with PRV JL-21 strain (MOI = 5) at 37°C for 1 hr and then washed three times with cold phosphate-buffered saline (PBS) to remove the unattached virus. The cells were cultured in DMEM with 2% FBS at 37°C in an incubator with 5% CO_2_ for 72 hr. Then, both infected cells and supernatants were collected and freeze-thawed three times. The PRV obtained from PK-15 cells was named PRV-PK. The PRV-PK was propagated on SH-SY5Y cells and passaged blindly for 20 generations. The PRV obtained from SH-SY5Y cells designated PRV-S1 to PRV-S20 for each generation, respectively. Then, the obtained PRV of the 20th passage (PRV-S20) alternately infected PK-15 cells and SH-SY5Y cells, respectively.

Human cells (including A549, WI38, HepG2, HEH2, HUVEC, HSAS4, SH-SY5Y, and HEK293), two permissive cells of PRV (Vero and ST), and two mouse cells (murine microglia (BV2) and mouse neuroblastoma-2a (N2a)) were infected with PRV for the indicated time. The copy numbers of PRV in the cells were evaluated by real-time PCR, and the cytopathic effect (CPE) was examined via a microscope (Nikon, Tokyo, Japan).

SH-SY5Y and HEK293 cells were cultured in the 3D cultures in ultralow attachment surface polystyrene 24-well plates which were individually transferred to 1.5 mL Eppendorf tubes, centrifuged at 8,000 rpm for 5 min, washed once with 500 *μ*L PBS, and then incubated with 100 *μ*L of DMEM/F12 or DMEM medium containing PRV-JL21 (MOI = 5) at 37°C for 2 hr with rotation. After that, the inoculum was removed by centrifugation at 8,000 rpm for 5 min, and the 3D cultures were washed twice with 500 *μ*L of PBS before being cultured in growth medium in ultralow attachment surface polystyrene 24-well plates. The proliferation of PRV in the 3D cells was evaluated by real-time PCR and Western blotting.

### 2.4. Quantum Dot (QD)-Conjugated PRV

For virus biotinylation, Vero cells were maintained in DMEM supplemented with 5% FBS and 0.02 g/L Biotinyl Cap PE (1,2-dioleoyl-sn-glycero-3-phosphoethanolamine-N-(cap biotinyl) (sodium salt), Aladdin, China) at 37°C in a 5% CO_2_ incubator to 80%–90% confluence. Cells were infected with PRV (MOI = 5). After adsorption for 1 hr, the cells were washed with PBS once and cultured in the fresh medium containing 2% FBS and 0.02 g/L Biotinyl Cap PE. When 80% of the cells showed cytopathic effect, the cells and culture medium were collected and freeze–thawed three times, followed by centrifugation at 1,000 rpm for 5 min to remove cell debris. Then, the supernatant was filtered using a 0.45 *μ*m polyethersulfone (PES) filter, and the filtrate was purified using the Universal Virus Concentration Kit (Beyotime, China). Finally, the biotinylated PRV was resuspended in PBS, stored at −80°C, or used immediately.

The water-soluble quantum dots (QDs) COOH with a maximal emission wavelength of 525 nm was purchased from Wuhan JiaYuan Quantum Dots Co. Ltd. (Wuhan, China). Then, QDs were incubated with biotinylated PRV overnight at 4°C in a mixer at a final concentration of 20 nM. After that, 4 mL of viral fluid was moved to the Amicon® Ultra-15 10K and centrifuged at 5,000 rpm for 45 min. The precipitate was resuspended with PBS, and the PRV-QDs were stored in a freezer at −80°C or used immediately.

### 2.5. Real-Time PCR

The virus genomic DNA was extracted using the Virus DNA/RNA Kit (Genfine, China) following the manufacturer's protocol. Then, real-time PCR analysis was performed on a Quantagene q225 real-time PCR system (Kubo Technology, China) using 2 × SYBR Green qPCR Master Mix (Bimake, China) with the PRV-SP-F/R primers. The amplification conditions were as follows: predenaturation at 95°C for 1 min; denaturation at 95°C for 15 s; annealing at 57°C for 15 s; and extension at 60°C for 30 s with a total of 40 cycles. Primers (PRV-SP-F: GGTTCAACGAGGGCCAGTACCG; PRV-SP-R: GCGTCAGGAATCGCATCACGT) were designed based on the PRV gD sequence (GenBank: OR228534) using PRIMER 5.0 and synthesized by Comate Bioscience Co., Ltd. (Jilin, China).

### 2.6. Western Blotting

Protein extracts were harvested from mock and virus-infected cells at 0–72 hours post infection (hpi) intervals. Briefly, cells were collected as described previously in Section 2.2. The collected cells were lysed with 50 *μ*L cell lysis buffer for Western and IP (Beyotime, Shanghai, China) on ice for 15 min, followed by centrifugation at 12,000 rpm, 4°C for 10 min. Then, the protein supernatant was transferred into a fresh tube and processed for Western Blotting.

Western blotting was performed according to the protocol described previously [26]. Briefly, protein samples were separated via 10% SDS-PAGE and electrotransformed onto the 0.45 *μ*m PVDF membrane. Then, the membrane was blocked with 5% skim milk for 2 hr at room temperature and incubated with anti-PRV gD monoclonal antibody (1 : 500, LvduBio, China) or anti-*β*-actin monoclonal antibody (1 : 10,000, Proteintech, Wuhan, China) at 4°C overnight. The membrane was incubated with HRP-labeled goat anti-mouse IgG (H + L) (1 : 5,000, WanleiBio, Shenyang, China) for 2 hr at room temperature. Subsequently, the protein band was developed using an ECL kit (WanleiBio, Shenyang, China) and examined via Bioanalytical Imaging System c600 (Azure Biosystems, Dublin, CA, USA).

### 2.7. Animal Experiment

Fifteen specific pathogen-free (SPF) Balb/c (6 weeks old and 20 g each) bred in an SPF environment were procured from Liaoning Changsheng Biotechnology Co., Ltd. (Liaoning, China). The mice were randomly divided into five groups, including the PRV-PK-infected group (three mice), PRV-S20-infected group (three mice), PRV-PK-QD-infected group (three mice), PRV-S20-QD-infected group (three mice), and mock group (three mice). Each group was housed separately and provided sterile food and water ad libitum. Before initiating the experiment, all mice were tested and confirmed negative for the following pathogens: porcine circovirus type 1 (PCV1), PCV2, PCV3, PCV4, porcine reproductive and respiratory syndrome virus (PRRSV), pseudorabies virus (PRV), classical swine fever (CSFV), porcine epidemic diarrhea virus (PEDV), transmissible gastroenteritis virus (TGEV), and porcine parvovirus (PPV).

For pathogenicity evaluation, mice were subcutaneously injected with 4 × 10^5^ TCID_50_ (in 50 *μ*L PBS, pH = 7.4) PRV-PK or PRV-S20. The control group was similarly inoculated with 50 *μ*L PBS (pH 7.4). At 10 days postinfection (dpi), the mice were euthanized humanely through intraperitoneal injection of sodium pentobarbital with a dose of 40 mg/kg body weight, followed by complete necropsy. Tissues, including the heart, liver, spleen, lung, kidney, and brain, were collected and fixed with 4% paraformaldehyde for hematoxylin and eosin (H&E) staining and immunohistochemical (IHC) assay.

For *in vivo* small animal imaging, mice were injected with QD-conjugated virus and examined by NightOWL II LB 983 small animal *in vivo* imaging system at 2, 6, and 10 dpi.

### 2.8. H&E and IHC Staining

Briefly, the tissue samples were fixed in 4% paraformaldehyde, followed by embedding in paraffin and sectioning. After that, the slides were evaluated using H&E staining and IHC assay.

For H&E staining, sections were dewaxed with graded xylenes and ethanols, stained with HE solution, dehydrated, and mounted with neutral resins. The slides were examined under a microscope (Nikon ECLIPSE E100, Tokyo, Japan) and analyzed using CaseViewer (3DHISTECH Ltd., Hungary).

For IHC staining, the sections were subjected to deparaffinization, antigen retrieval, and blocking with albumin from bovine serum (BSA). Then, slides were stained with anti-PRV gD monoclonal antibody (1 : 100, LvduBio, China) overnight at 4°C and washed with PBS three times. After being washed, the slides were incubated with HRP-conjugated goat antimouse IgG (H + L) (1 : 200, Servicebio, China) at 37°C for 1 hr and then washed with PBS three times. Then, the slides were treated with 500 *μ*L diaminobenzidine (DAB) solution (Servicebio, China) and washed with ddH_2_O. Afterward, the slides were counterstained with hematoxylin, examined by a microscope (Nikon ECLIPSE E100, Tokyo, Japan), and analyzed using CaseViewer (3D HISTECH Ltd., Hungary).

### 2.9. Isolation of PRV from Mice Tissue

The tissue was homogenized and resuspended with PBS, followed by three freeze–thaw cycles. After centrifugation at 5,000 rpm for 10 min, the supernatant was incubated with 2 mg/mL streptomycin and 2,000 U/mL penicillin at 4°C for 24 hr and filtered with a 0.22 *μ*m PES filter. Then, PK-15 cells at 80% confluent were inoculated with the filtrate at 37°C for 1 hr, followed by washing the unbound virus with PBS. Then, the cells were cultured in prewarmed DMEM (Gibco, USA) containing 2% FBS and 1% agarose at 37°C in a 5% CO_2_ incubator. After that, plaques were picked and seeded into new cell culture plates for further virus isolation and purification. The PRV obtained was designated PRV-m.

### 2.10. Sequence Alignment

The sequence information of *gD*, *gB*, *gH*, *gL*, *TK*, and *UL41* genes of PRV-JL21 was submitted to GenBank (GenBank Nos. OR228534, OR195723, OP922505, OP972924, OR195724, and OP168821). The *gD*, *gB*, *gH*, *gL*, *TK*, and *UL41* of PRV-PK (PRV JL-21 strain) were used as the reference sequence, and multiple alignments were performed using Multalign (http://multalin.toulouse.inra.fr/multalin/multalin.html, accessed on 2023/09/08).

### 2.11. Transmission Electron Microscope Assay

Cells infected with PRV were collected and lysed by three freeze–thaw cycles. Then, the cell lysate was centrifuged at 3,000 rpm for 20 min, followed by centrifugation at 10,000 rpm for 20 min to remove the cellular proteins and media components. After that, the supernatant was ultracentrifuged at 30,000 rpm for 2 hr in a Beckman SW50.1. The precipitate virions were collected, and purified virions were stained with 2% uranyl acetate and examined at 80 kV on the Hitachi TEM system HC-1 (Hitachi, Japan).

### 2.12. Statistical Analysis

The statistical analysis was conducted using GraphPad 9.0 software. Differences between the control and experimental groups were assessed using the one-way analysis of variance (ANOVA) or two-way ANOVA. Three independent experiments present data as mean ±  standard deviation (SD). A *p value* of less than 0.05 was considered statistically significant:  ^*∗*^, *p* < 0.05;  ^*∗∗*^, *p* < 0.01; and  ^*∗∗∗*^, *p* < 0.001.

## 3. Results

### 3.1. PRV Is Infectious, Proliferative, and Pathogenic in Human Cells

**Figure 1:**
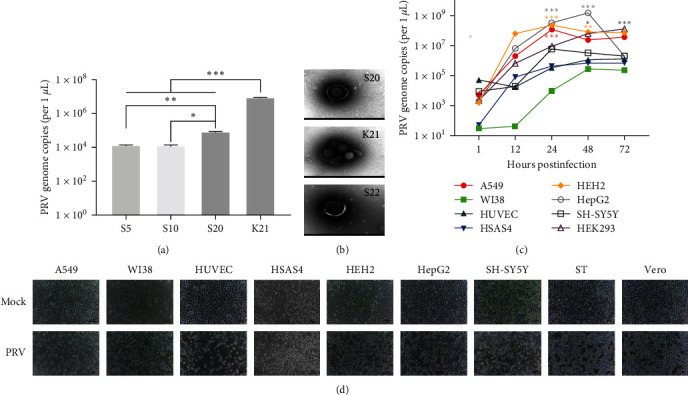
PRV is infectious, proliferative, and pathogenic in human cells. Porcine-derived PRV (PRV-PK) was blind passaged in human neuroblastoma cells (SH-SY5Y) 20 times (S1 to S20), followed by transferring the virus (PRV-S20) to PK-15 cells. Then, the human-adapted PRV of the 20th passage (PRV-S20) alternately infected PK-15 and SH-SY5Y cells, respectively. S5, S10, S20, and S22 refer to the 5th, 10th, 20th, and 22nd generations of PRV passaged blindly in human SH-SY5Y cells. K21 means the PRV isolated from PK-15 cells, which was infected with the 20th passage of PRV from SH-SY5Y (PRV-S20). (a) Copy numbers of different passaged PRVs. (b) TEM. Bar = 200 nm. (c) One-step growth curve. Different human cancer cells (A549 and HepG2) and human normal cells (WI-38, HUVEC, HSAS4, HEH2, SH-SY5Y, and HEK293) were infected with human-derived PRV (PRV-S20) for 1, 12, 24, 48, and 72 hr, followed by evaluation of the viral genomic copies. (d) CPE caused by human-adapted PRV (PRV-S20). Different cells were infected with human-adapted PRV (PRV-S20) for 72 hr and then examined using a microscope (×40).  ^*∗*^*p* < 0.05;  ^*∗∗*^*p* < 0.01;  ^*∗∗∗*^*p* < 0.001.

To evaluate whether PRV is infectious in human cells, the human neuroblastoma cell line (SH-SY5Y) was infected with PRV (PRV-PK) for 72 hr, followed by blind passage of the virus in SH-SY5Y cells 20 times. Then, the obtained PRV of the 20th passage (PRV-S20) alternately infected PK-15 cells and SH-SY5Y cells, respectively. As shown in Figure 1(a), the copy numbers of PRV remained stable at 10^4^−10^5^ copies/*μ*L in human cells. Notably, a significant increase in PRV copies can be achieved in PK-15 cells infected with the PRV-S20. Furthermore, the mature PRV virus particles can be detected in SH-SY5Y cells (Figure 1(b)). The morphology of the human cell-adapted PRV is similar to that of the PRV isolated from PK-15 cells. These results suggest that the human cells are permissive for PRV infection. PRV can effectively infect and replicate in human cells, and the progeny virus in human cells is still infectious.

Because PRV can replicate stably in SH-SY5Y cells and PRV has been detected in human patients in previous reports, it is necessary to analyze further whether PRV is pathogenic in human cells. Therefore, human cells, including A549, WI38, HepG2, HEH2, HUVEC, HSAS4, SH-SY5Y, and HEK293, were infected with PRV-S20 for 72 hr. As shown in Figure 1(c), copy numbers of PRV-S20 increased in a time-dependent manner, indicating the virus can also replicate in these human cells, including human cancer cells and normal cells. Moreover, the CPE caused by human cell-adapted PRV (PRV-S20) in human cells was similar to that of the virus caused in its permissive cells, ST and Vero (Figure 1(d)).

**Figure 2:**
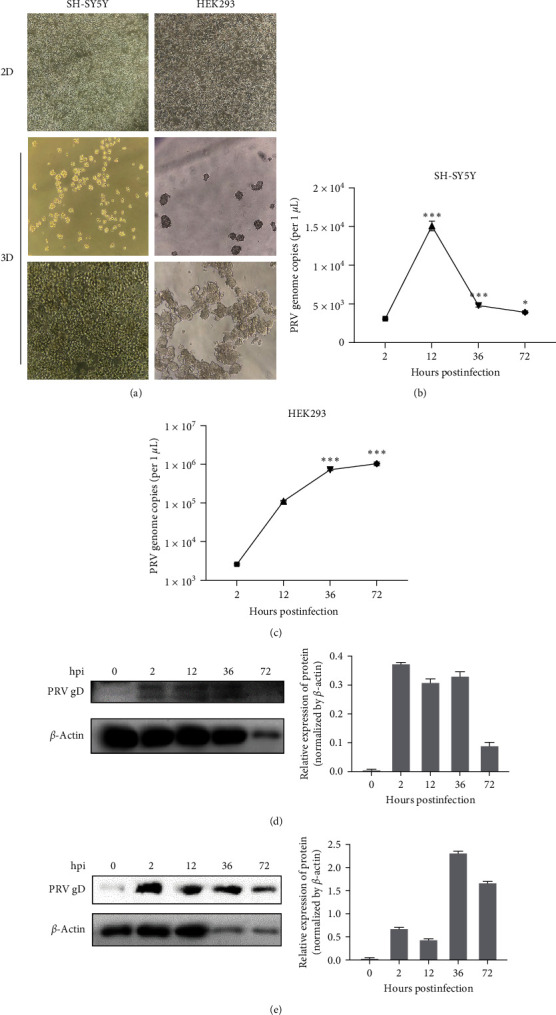
PRV can infect and proliferate in 3D-cultured human cells. (a) The cell morphology of SH-SY5Y and HEK293 cultured in 2D plates or 3D ultralow attachment plates. For 3D culture, low (top panel) and high (bottom panel) confluency cells were used. (100x) (b and c) One-step growth curve of PRV. 3D cultures of SH-SY5Y (b) and HEK293 (c) were infected with PRV-JL21 for 2, 12, 36, and 72 hr, followed by the evaluation of PRV genome copies using real-time PCR. (d and e) Western blotting was performed to examine the expression of PRV glycoprotein D (gD) in the 3D cultures of SH-SY5Y (d) and HEK293 (e) at intervals of 0–72 hpi. Anti-PRV gD monoclonal antibody and anti-*β*-actin monoclonal antibody were used as the primary antibody, and HRP-conjugated goat antimouse IgG (H + L) was used as the secondary antibody. Western blots (d and e) were quantified in the left panel by grayscale analysis with normalization to *β*-actin using ImageJ version 1.8.3.  ^*∗*^*p* < 0.05;  ^*∗∗∗*^*p* < 0.001.

3D spheroids are widely used in multicellular 3D models, which provide a more physiologically relevant model and can successfully simulate the microenvironment of various tissue types in disease states compared to 2D cell cultures [27, 28]. Therefore, 3D multicellular spheroids were further developed using SH-SY5Y and HEK293 cell lines to evaluate the infectious characteristics of PRV in human cells. As shown in Figure 2(a), human cells SH-SY5Y and HEK293 can effectively form 3D spheroids. When the spheroids were infected with PRV, copy numbers of PRV genomic DNA significantly increased compared with the 2 hpi, indicating a replication of the viral genomic DNA during the infection in the spheroids (Figures 2(b) and 2(c)). These results were further confirmed by evaluating the viral glycoprotein D (gD) levels in the infected spheroids using Western blotting (Figures 2(d) and 2(e)), indicating that 3D spheroids derived from both human cell types are susceptible to infection by the PRV-JL21.

Besides, PRV genes, including *gD*, *gB*, *gH*, and *gL*, encode important surface glycoproteins, and the *TK* and *UL41* genes are related to PRV virulence. The sequences of these genes of PRV-S20 were aligned with that of the porcine cell-derived PRV (PRV-PK). As expected, the sequences of the genes mentioned above (GenBank Nos. OR228534, OR195723, OP922505, OP972924, OR195724, and OP168821) of the two viruses are identical.

These results indicate that PRV is contagious, proliferative, and pathogenic in human cells and has the potential to infect humans and cause diseases.

### 3.2. PRV Adapted to Human Cells Has Similar Characteristics to PRV from Pigs

**Figure 3:**
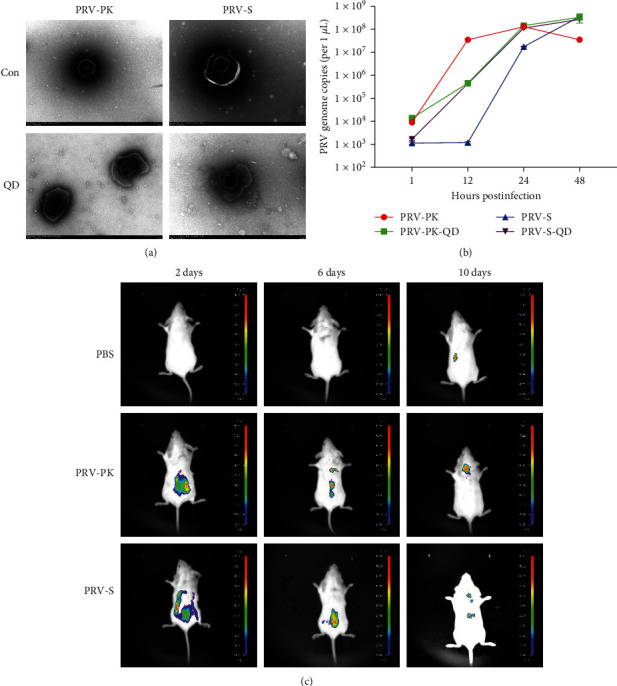
Characteristics of quantum dot labeled PRV. PRV-PK, PRV derived from porcine; PRV-S, PRV adapted to human cells. QD, quantum dot. (a) TEM of PRV labeled with or without QDs. Bar = 200 nm. (b) One-step growth curve of PRV labeled with or without QDs. (c) Real-time tracking of QD-labeled PRV in mice.

The virus was labeled with QDs and analyzed *in vitro* and *in vivo* to evaluate further the characteristics of PRV adapted to human cells. As shown in Figure 3(a), the PRV labeled with QDs had a similar morphology to the wild-type (WT) ones. Furthermore, the one-step growth curve demonstrated that the QD-labeled PRV (PRV-PK-QD and PRV-S20-QD), as well as the WT virus (PRV-PK and PRV-S20), can effectively replicate in PK-15 cells (Figure 3(b)). Moreover, all the PRV, including PRV derived from porcine (PRV-PK) and PRV adapted to human cells (PRV-S20), could infect mice and spread *in vivo* (Figure 3(c)). Although the fluorescence intensity gradually weakened with time in the infected mice, it gradually migrated to the brain, suggesting that the PRV is a brain tropism virus. These results indicate that PRV adapted to human cells has characteristics similar to those of PRV isolated from pigs.

### 3.3. PRV Adapted to Human Cells Is as Pathogenic as That of PRV from Pigs in Mice

**Figure 4:**
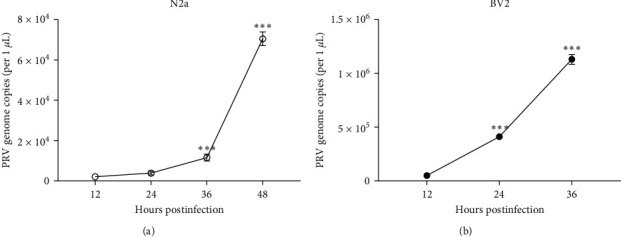
One-step growth curve of PRV in mice cells. Murine microglia (BV2) and mouse neuroblastoma-2a (N2a) cells were infected with PRV-S20, followed by real-time PCR of virus copy numbers at 12, 24, 36, and/or 48 hpi (a) and (b).  ^*∗∗∗*^*p* < 0.001.

**Figure 5:**
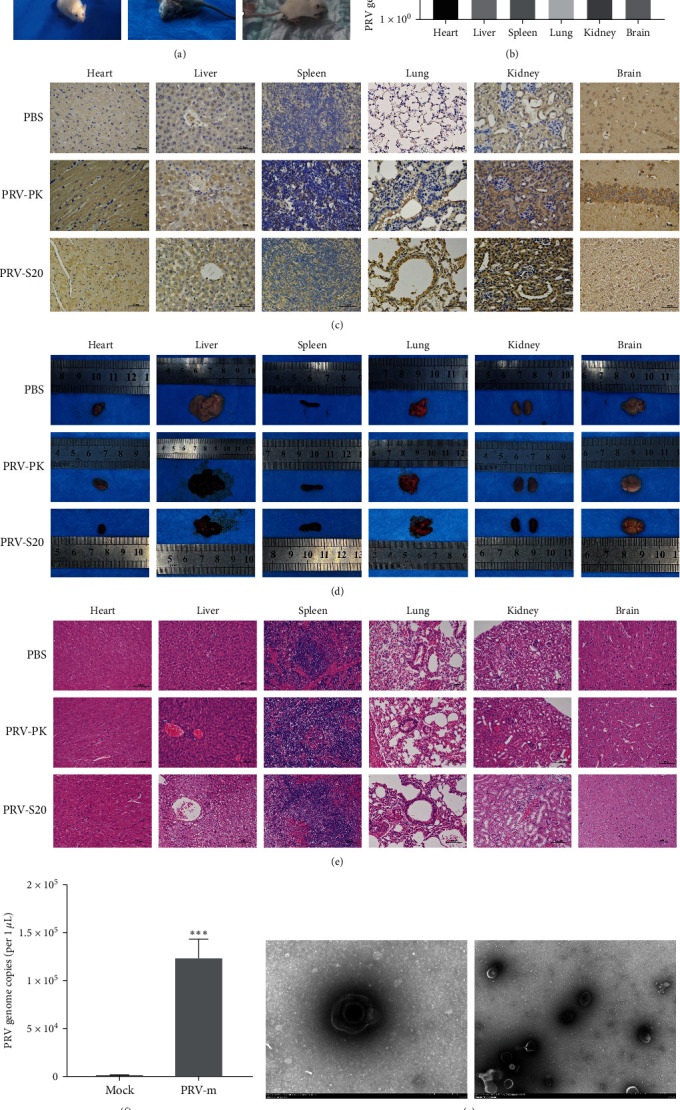
PRV adapted to human cells is as pathogenic as that of PRV from pigs in mice. Mice were treated with PBS or infected with PRV-PK or PRV-S20 for 5 days. The physiological status of mice was recorded on the fifth day after the treatment. At the same time, the mice of all groups were euthanized on the same day, and the collected tissues were further analyzed. (a) Physiological status of mice. (b) Copy numbers of PRV from various tissues of the PRV-S20 infected mice. (c) IHC staining. Anti-PRV gD monoclonal antibody was used as the primary antibody, and HRP-conjugated goat antimouse IgG (H + L) was used as the secondary antibody. (d) Necropsy results of different groups. (e) HE staining. (f) Copy numbers of PRV (PRV-m) isolated from the mice infected with PRV-S20. (g) TEM of PRV (PRV-m) isolated from the mice infected with PRV-S20. Bar = 200 nm (top panel) or 1 *μ*m (bottom panel).  ^*∗∗∗*^*p* < 0.001.

To evaluate the characteristics of PRV, murine microglia (BV2) and mouse neuroblastoma-2a (N2a) cells were infected with PRV adapted to human cells (PRV-S20). As a result, the copy numbers of PRV increased in a time-dependent manner in both cells. Significantly, the copy numbers of PRV began a substantial increase from 12 hpi and a significant enhancement at 36 hpi in N2a cells and 24 hpi in BV2 cells compared with that of the 12 hpi (Figure 4), suggesting that the PRV-S20 is replicative in mice neurocytes. Therefore, we infected the mice with PRV-S20 and PRV-PK for further analysis (Figure 5). As shown in Figure 5(a), the mice in the control group showed healthy signs. In contrast, the mice in the virus-infected group showed symptoms such as depression, loss of appetite, and accelerated breathing before death. Some mice in the virus-infected group died shortly after typical PRV symptoms, such as itching, hunchback, and biting at the injection site.

Furthermore, the mice of all groups were euthanized on the fifth day after the treatment, and the collected tissues were further analyzed. The genome of PRV (Figure 5(b)) and PRV antigen (Figure 5(c)) can be detected in the heart, liver, spleen, lung, kidney, and brain of the infected mice, suggesting that PRV-S20 was widely distributed in different tissues of the infected mice.

Moreover, the autopsy revealed that the heart, liver, lungs, and kidneys of the mice had varying degrees of hyperemia. An enlarged spleen was present, and no apparent lesions were observed in the brain (Figure 5(d)). The results of HE showed that the myocardial cells in PRV-PK and PRV-S20 infected groups showed coagulation necrosis in different degrees (Figure 5(e)). Liver cells were arranged in disorder, with nuclear condensation, cytoplasm melting, unclear boundary, and inflammatory lymphocytic infiltration in the portal area. Pulmonary interstitial hemorrhage, fibrous hyperplasia, inflammatory lymphocytic infiltration, and partial alveolar expansion or atrophy occurred in lung tissue. There was inflammatory lymphocytic cell infiltration in the renal interstitium, glomerular lobulation, and renal tubular loss of typical structure, leading to vacuolar changes. Few cells in the renal corpuscles were irregular in shape and had glomerular capsule adhesion. Besides, neurons showed vacuolar changes and cytolysis in PRV-S20 infected groups, suggesting degeneration and necrosis occurred in neurons. Small intestinal glands decreased, the continuity and integrity of mucosal epithelium were destroyed, and inflammatory cell clusters and crypt cysts appeared. These results indicate that different degrees of pathological changes occurred in the heart, liver, spleen, lung, kidney, brain, and intestine of the mice infected with PRV-PK and PRV-S20. PRV-S20 has infectivity and pathogenicity in mice.

Besides, a PRV was isolated from mice infected with PRV-S20 and designated PRV-m (Figure 5(f)). The PRV-m can replicate in PK-15 cells with about 1.2 × 10^5^ copies/*μ*L. The sequences of *gB*, *gD*, *gH*, *TK*, and *UL41* genes of PRV-m were consistent with those of the PRV-S20 and PRV-PK. Typical PRV virions can be observed by electron microscope (Figure 5(g)). These results demonstrate that PRV-S obtained from human cells maintains PRV's infectious and pathogenic characteristics, further confirming that human cells are permissive for PRV infection.

## 4. Discussion

Previous studies have detected PRV in patients, suggesting it may be a pathogen responsible for the disease [13, 18, 19, 20, 21, 22, 23, 24]. It was reported that PRV gD potentially binds to human nectin-1 [22]. We previously found that PRV gD may also bind to human nectin-1, nectin-2, and heparan sulfate (HS) [29]. These results imply that PRV has the potential to infect humans. Moreover, it has been ascertained that PRV can infect mammals, including cats, dogs, and cattle [13]. These cases offer new insights for further exploring the transmission and pathogenic mechanisms of PRV in mammals. However, whether PRV can infect humans or belongs to a zoonotic virus is still controversial. In this study, we continuously passaged PRV in human nerve cells and obtained progeny viruses, designated PRV-S (Figure 1(a)), which has a similar morphology to that of the PRV isolated from PK-15 cells (Figure 1(b)). Furthermore, the PRV-S can replicate in numerous human cells, including cancer cells and normal cells, with an increased copies number in a time-dependent manner (Figure 1(c)). Moreover, when infected with human cell-adapted PRV-S20, several cells, including Vero, ST, PK15, and various human cell lines, also exhibited typical pathological changes (Figure 1(d)). Besides, 3D spheroids derived from both human cell types are susceptible to infection by the PRV-JL21 (Figure 2). Notably, the copy numbers of PRV genome increased in a time-dependent manner in the spheroids generated from HEK293. In contrast, it was increased at 12 hpi and decreased after that in the SH-SY5Y spheroids. Possible reasons are that (1) the SH-SY5Y cell is a human neuroblastoma cell, which is more permissive to PRV than the human embryonic kidney cell HEK293; (2) the tolerance of SH-SY5Y cells to the acidic environment in the culture medium is lower than that of HEK293 cells; thus SH-SY5Y cells in a pathological state lead to the decrease of PRV proliferation in the late stage of the infection. These results indicate that the progeny PRV obtained by proliferating PRV in human cells exhibits infectivity and pathogenicity in other types of cells, including human, porcine, and monkey cells. This finding further confirms that the human cell-adapted PRV can infect human cells and cause related diseases.

Furthermore, given the high homology between mouse and human proteins, we established a PRV infection model using Balb/c mice. The results showed that the PRV-S20 was neurotropic and could cause pathological damage to the internal organs and brain of mice (Figure 3(a)). Using QD-conjugated PRV to infect mice, we tracked the movement of the virus in mice. The results showed that QD-labeled PRV (PRV-PK and PRV-S20) migrated to the brain region in the late stages of infection (Figure 3(c)). This suggests that the human cell-adapted PRV-S20 possesses brain tropism, which is a typical characteristic of herpesvirus [5, 30,31,32]. Moreover, the results of IHC and H&E also demonstrated that PRV-S20 adapted to human cells was distributed in various mouse internal organs and caused varying degrees of damage in the heart, liver, lung, kidney, and brain (Figure 5). These results suggest that PRV can propagate in human cells and generate offspring with infectivity and pathogenicity to mice.

Interestingly, when passaged blindly for 20 generations in SH-SY5Y cells, the copy numbers of the virus were approximately 10^4^ ~ 10^5^  copies/*μ*L at the 5th, 10th, 20th, and 22nd generations. However, when PK15 cells were infected with PRV-S20, the copy numbers of PRV increased significantly, with approximately 5 × 10^6^ copies/*μ*L. This result indicates that PRV exists in the neuronal cell line SH-SY5Y with a lower copy number but maintains its infectivity in the cells. When it returns to susceptible cells, such as PK15, it can rapidly increase and reach a higher viral load. This result is also consistent with the infection characteristics of herpesvirus, which is latent in the nervous system and invades epithelial tissue when the host is stimulated, or the immune system is weakened and proliferates rapidly, thus causing diseases to varying degrees [5, 33, 34, 35]. Moreover, the gene sequences of the critical glycoproteins gD, gB, gH, gL, and the pathogenicity protein TK of PRV-S20 were highly similar to those of the parental virus PRV-PK. PRV-S20 also exhibited infection characteristics and pathogenicity identical to the parental virus PRV-PK. This result suggests that the infectivity of PRV obtained from human cells is an inherent property rather than the result of selective pressure for adaptation to human cells.

Given the infection characteristics of herpesviruses and the potential of PRV to infect humans [15, 36], we assumed that PRV can also be stably passaged in human neural cells and produce progeny virus with infection and pathogenicity. To test our hypothesis, we used PRV-PK to infect SH-SY5Y cells to investigate whether PRV can indeed stably transmit in human neural cells. The progeny viruses obtained were subjected to cell and animal experiments to assess their infection and pathogenicity. These results are consistent with Koch's postulates that the pathogen can infect the host, be isolated from the host, and infect it again. The findings in this study supported our hypothesis, indicating that the cross-species progeny PRV can still infect the original host cells and other human cells, causing cell lesions. This infection characteristic is an inherent attribute rather than being selected under selective pressure. Therefore, it can be concluded that PRV has the potential for cross-species transmission, which can infect pigs, humans, and other animals and cause severe clinical symptoms in the infected individual.

This study demonstrates that PRV can cross-transmit in human cells and animal models and *vice versa*. However, lacking human cases makes it difficult to establish the infection and transmission of PRV in humans. Therefore, further study must investigate the PRV infection mechanism in human cells.

## 5. Conclusions

Overall, this study confirmed that PRV has the potential to cross-species transmission and infection in humans and *vice versa*, resulting in different degrees of diseases. These results can provide references for developing more effective prevention and control measures, preventing the spread of related epidemics, and promoting the development of public health.

## Data Availability

All data generated or analyzed during this study are included in this published article.
